# Dasiglucagon in Children With Congenital Hyperinsulinism Up to 1 Year of Age: Results From a Randomized Clinical Trial

**DOI:** 10.1210/clinem/dgae818

**Published:** 2024-11-25

**Authors:** Diva D De Leon, Indraneel Banerjee, Sebastian Kummer, Sune Birch, Eva Bøge, Jelena Ivkovic, David M Kendall, Paul S Thornton

**Affiliations:** Congenital Hyperinsulinism Center, Division of Endocrinology and Diabetes, The Children's Hospital of Philadelphia, Philadelphia, PA 19104, USA; Department of Pediatrics, Perelman School of Medicine at the University of Pennsylvania, Philadelphia, PA 19104, USA; Department of Paediatric Endocrinology, Royal Manchester Children's Hospital, Manchester, M13 9WL, UK; Department of General Paediatrics, Neonatology and Paediatric Cardiology, Medical Faculty and University Hospital Düsseldorf, Heinrich Heine University Düsseldorf, Düsseldorf, 40225, Germany; Zealand Pharma A/S, Søborg, 2860, Denmark; Zealand Pharma A/S, Søborg, 2860, Denmark; Zealand Pharma A/S, Søborg, 2860, Denmark; Zealand Pharma A/S, Søborg, 2860, Denmark; Congenital Hyperinsulinism Center, Department of Endocrinology, Cook Children's Medical Center, Fort Worth, TX 76104, USA

**Keywords:** congenital hyperinsulinism, dasiglucagon, hypoglycemia, treatment

## Abstract

**Context:**

Congenital hyperinsulinism (CHI) is a cause of persistent hypoglycemia in childhood with a considerable risk of lifelong neurological sequelae. Available pharmacological therapies are limited. Dasiglucagon is a glucagon analog for the treatment of hypoglycemia.

**Objective:**

To assess the efficacy and safety of dasiglucagon in children with CHI up to 1 year of age.

**Methods:**

This study included a randomized, crossover, double-blind, placebo-controlled part 1 and an open-label, single-arm part 2 at 4 centers in Germany, the United Kingdom, and the United States. Participants comprised children with CHI aged 7 days to 12 months who were dependent on IV glucose. In part 1, participants were randomized to dasiglucagon or placebo for 48 hours, then crossed over to the other treatment for 48 hours. In part 2, all participants received dasiglucagon for 21 days. The primary outcome was mean IV glucose infusion rate (GIR) in the last 12 hours of part 1.

**Results:**

Between June 19, 2020, and February 9, 2022, 12 eligible participants were randomized to dasiglucagon–placebo (n = 7) or placebo–dasiglucagon (n = 5). The IV GIR was significantly reduced with dasiglucagon compared with placebo (least-squares mean 4.3 mg/kg/min [95% confidence interval [CI], 1.04 to 7.60 mg/kg/min] and 9.5 mg/kg/min [95% CI, 6.24 to 12.81 mg/kg/min], respectively; *P* = .004). The most frequent adverse events in both treatment groups were gastrointestinal, dermatological, and metabolism and nutritional disorders.

**Conclusion:**

In infants with CHI, dasiglucagon significantly reduced the amount of IV glucose needed to maintain euglycemia compared with placebo. Dasiglucagon represents a promising treatment for the management of CHI.

Congenital hyperinsulinism (CHI) is the most common cause of severe and persistent hypoglycemia in early childhood characterized by dysregulated insulin production by pancreatic β cells, resulting in excess insulin secretion independent of plasma glucose (PG) (1). Several pathogenic gene variants have been described (2-4), with histopathological subtypes of focal, diffuse, and atypical CHI (1). Infants with CHI are at substantial risk of long-term neurodisability from hypoglycemia-induced brain injury. Therefore, early diagnosis and treatment are critical (1, 5, 6).

Most children with CHI present shortly after birth with severe hypoglycemia and high glucose requirements, necessitating IV dextrose. Pharmacological options for the management of severe CHI are limited. Diazoxide is the only approved therapy for CHI, with off-label use of somatostatin analogs and sirolimus frequently complicated by major side effects (1, 7). Many children with CHI are unresponsive to existing therapies and continue to experience hypoglycemia or require burdensome nutritional support (1). In severe cases, subtotal/near-total pancreatectomy may be required, associated with postsurgical insulin-requiring diabetes mellitus and exocrine pancreatic insufficiency (1, 8).

Dasiglucagon (9) is a modified glucagon analog, stable in aqueous solution without fibrillation or aggregation, unlike standard glucagon therapy (10). It is approved for the treatment of severe hypoglycemia in people living with diabetes aged over 6 years in the United States (9) and was granted orphan drug designation for the treatment of CHI in the United States and EU in 2017 (11, 12).

This phase 2/3 study aimed to investigate the efficacy and safety of individually titrated, continuous dasiglucagon subcutaneous (SC) infusions to reduce hypoglycemia in infants aged 7 days to 12 months with severe CHI who were dependent on IV dextrose support. The current study is part of a wider clinical development program for dasiglucagon in CHI, which also includes a previously published study on the efficacy and safety of dasiglucagon in infants and children aged 3 months to 12 years (ClinicalTrials.gov identifier NCT03777176) (13), in addition to an ongoing long-term safety and efficacy study (ClinicalTrials.gov identifier NCT03941236), in which participants of the first 2 studies were enrolled.

## Materials and Methods

### Study Design

**Figure 1.**
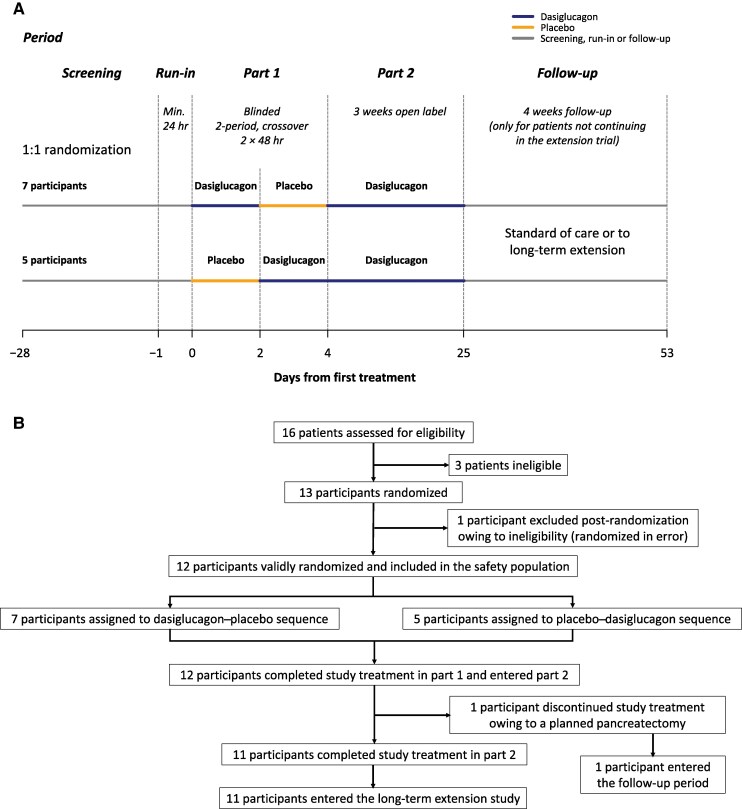
Study design scheme (A) and participant flow diagram (B).

Abbreviation: min., minimum.

The trial was a combined phase 2/3 multicenter clinical study in 2 parts: a randomized, crossover, double-blind, placebo-controlled part 1 with 2 periods (48 hours each) and an open-label, single-arm part 2 (21 days) (Fig. 1A). The treatment period was limited to 48 hours because CHI carries the risk of neurodevelopmental sequelae if treatment is delayed. Therefore, exposure to placebo was restricted to the minimum period necessary to demonstrate treatment effect. The study was conducted at 4 centers specializing in CHI (1 site each in Germany and the United Kingdom, 2 sites in the United States). The study was registered with the EU Clinical Trials Register (2017-004545-24) and with ClinicalTrials.gov (NCT04172441).

The study protocol was individually approved by independent ethics committees and institutional review boards at each of the study centers. All participants’ legally authorized representatives provided written informed consent. The study was conducted in accordance with International Council for Harmonisation of Technical Requirements for Pharmaceuticals for Human Use Good Clinical Practice and the principles of the Declaration of Helsinki, as well as other applicable local ethical and legal requirements.

### Participants

Eligible participants were aged 7 days to 12 months (inclusive) at screening, with a body weight of at least 4.4 lb (2.0 kg) and diagnosed with CHI. Criteria for CHI diagnosis included the presence of elevated insulin with suppressed free fatty acids (<1.7 mmol/L) and β-hydroxybutyrate (<1.8 mmol/L) and/or a glycemic response to glucagon (an increase in PG of >30 mg/dL [1.7 mmol/L] after 1 mg IV or intramuscular glucagon administration). All participants were dependent on continuous IV glucose through a central venous catheter at the time of screening and at randomization. Patients were excluded if transient CHI was anticipated (eg, transient hyperinsulinism due to maternal diabetes or perinatal stress); the baby was born preterm (less than 34 weeks); or the baby had known/suspected severe brain damage, hypertension/hypotension, or metabolic, endocrine, or syndromic causes of hypoglycemia without hyperinsulinism. Additional exclusion criteria included a mean glucose infusion rate (GIR; dextrose content in IV fluid infusion expressed in mg/kg/min) below 10 mg/kg/min to maintain glucose above 70 mg/dL (3.9 mmol/L) or use of glucagon or additional enteral glucose in the 24 hours before randomization.

### Randomization and Masking

In part 1, all participants were treated with dasiglucagon and placebo in randomized (1:1), double-blind, crossover treatment sequences, with treatment order assigned by an automated web-response system. The randomization list was created before study initiation by an independent statistician, based on randomized block sizes of 4 and stratified by region (United States/Europe). Investigators were not aware of the block size of the randomization scheme. Dasiglucagon and placebo were supplied in identical vials to mask treatment assignment.

### Procedures

The principal investigator was responsible for the recruitment and conduct of the trial at each study site. Investigators, parents, and carers were subject to intervention blinding. Blinded continuous glucose monitoring (CGM) without alarm function was initiated 24 hours before randomization (Dexcom G4 or G6 system, Dexcom Inc., San Diego, CA) and continued through part 2. During IV glucose administration, PG was measured hourly by a StatStrip Xpress2 PG meter (Nova Biomedical, Waltham, MA). Eligible participants completed a 24-hour run-in period to confirm the GIR randomization requirement of at least 10 mg/kg/min. All carbohydrate supplementation and fortification of feeds were prohibited during this period.

Dasiglucagon (4 mg/mL) and placebo were provided in vials for SC administration through an Accu-Chek Combo infusion pump, using the Accu-Chek Spirit 3.15 mL cartridge system and the Accu-Chek FlexLink or Accu-Chek Rapid-D Link infusion set (Hoffman-La Roche AG, Basel, Switzerland). Following randomization, the participants received an SC infusion of either dasiglucagon or placebo for 48 hours (part 1, period 1), then crossed over to the opposite treatment for an additional 48 hours (part 1, period 2) (Fig. 1A). Dasiglucagon treatment was initiated at 10 μg/hour. Every 2 hours, the infusion rate was increased in 10 μg/hour increments until 1 of the following occurred: the participant weaned off IV glucose; PG over the previous 2 hours was constantly above 120 mg/dL (6.7 mmol/L); IV GIR did not decrease despite 2 sequential dose increments; the maximum infusion rate of 70 μg/hour was reached; or emerging adverse events (AEs) were judged by the investigator to be causally related to study treatment and limited further dose escalation. The IV GIR was reviewed, evaluated, and adjusted (if indicated) every hour according to a protocol-specified, PG-driven algorithm to maintain PG above 70 mg/dL. Participants remained in the hospital for the duration of part 1. During part 1, daily study assessments included the collection of data on weight, length, vital signs, physical and age-appropriate neurological examinations, electrocardiograms, concomitant medications, safety, local tolerability, and blood antidrug antibodies (ADAs, predose).

Following part 1, participants continued in open-label part 2, with the opportunity to continue dasiglucagon for an additional 21 days (Fig. 1A). Dasiglucagon dosing was initiated at 10 μg/hour and IV GIR was adjusted to match the rate at the end of the run-in period. During part 2, the dasiglucagon infusion rate, up to a maximum infusion rate of 70 μg/hour, and the amount and route of supplemental carbohydrates were adjusted at the investigator's discretion (no guidelines applied). Additional CHI treatments were permitted if further uptitration of dasiglucagon was not possible due to AEs or if the maximum infusion rate (70 μg/hour) was reached. Throughout part 2, participants had the same study assessments as performed during part 1 on days 5, 6, 11, 18, and 25. In addition, blood samples were obtained on days 5, 6, and 25 for hematology, biochemistry, pharmacokinetics and on day 25 for ADA assessments. Participants could be discharged to continue home treatment after weaning off IV glucose and meeting local study-site discharge criteria, contingent on home administration of treatment via a pump, use of CGM or self-monitored PG at least 3 times daily, and study center attendance for assessments. At home, parents/guardians recorded carbohydrate supplements, hypoglycemic and safety events, medications, and health resource utilization in a diary. Participants who were discharged from the hospital before day 25 were contacted by the investigator by telephone the day after discharge for safety assessments.

At study completion, participants were offered entry into a long-term extension study (ClinicalTrials.gov NCT03941236, EU Clinical Trials Register identifier 2017-004546-15). The opportunity for the participants to enter the long-term extension study at the end of part 2 was based on the investigator's confirmation of a continued positive benefit-risk balance.

### Outcomes

The primary outcome was the mean IV GIR in the last 12 hours of each 48-hour treatment period in part 1. The key secondary efficacy outcome was the total amount of carbohydrates (grams) administered (regardless of route) per day in part 1. Additional secondary outcomes from part 2 were time to complete weaning off IV glucose (for a continuous period of at least 12 hours), time to hospital discharge, time to pancreatectomy, and time in hypoglycemia (PG below 70 mg/dL) or clinically significant hypoglycemia (PG below 54 mg/dL [3.0 mmol/L]), detected by CGM. The time to complete weaning off IV glucose was defined as the time from first dasiglucagon exposure during part 2 to the first point in time when the participant was off IV glucose for a continuous period of at least 12 hours. The time to hospital discharge was defined as the time from the first dasiglucagon exposure in part 2 to discharge from the hospital. Time to pancreatectomy was defined as the time from the first dasiglucagon exposure in part 2 to near-total pancreatectomy, defined as removal of at least 95% of the pancreas.

Safety outcomes included AEs, serious AEs, AEs of special interest (AESIs), changes in clinical evaluations (vital signs, physical examinations, 12-lead electrocardiogram), and laboratory assessments (hematology, biochemistry, ADAs). AESIs comprised the following adverse events: suspicion of necrolytic migratory erythema (NME; to be reported in case of any skin eruption lasting more than 48 hours, where NME could not be excluded), risk of liver injury, loss of consciousness, partial and generalized seizures, and clinically significant changes in blood pressure or heart rate. Suspicion of NME was assessed based on therapeutic tests with topical treatments, blood tests, therapeutic tests with lowering or pausing the dasiglucagon infusion rate, and dermatologist consultation, including possible skin biopsy.

### Statistical Analysis

To demonstrate treatment effect, we undertook a power calculation to determine the minimum number of participants required to meet the primary endpoint while at the same time minimizing the number of participants who received the placebo. It was calculated that 12 participants would be required to receive dasiglucagon or placebo 1:1 to give 89% power (1-sample *t*-test) to detect a GIR reduction for dasiglucagon after 48 hours of at least 7.5 mg/kg/min (SD, 7.36) compared with placebo, based on a reference study (14). The weighted mean IV GIR and total amount of carbohydrates administered per day were analyzed in the full analysis set (FAS) using a mixed multivariable regression model including treatment and period as fixed effects and patient as random effect to account for correlated measures. Period effects are common and included for statistical efficiency. The 2-sided 95% confidence interval (CI) for the treatment difference was calculated from the mixed regression model. Statistical significance testing was 2-sided, with a significance level of α = .05. Control of the type I error rate for primary and key secondary endpoints was assessed using a fixed-sequence hierarchical testing strategy at the same significance level (α = .05, 2-sided test). Statistical analyses were run using SAS software version 9.4 (SAS Institute Inc., Cary, NC).

Continuous outcomes, including total amount of carbohydrates administered, percentage of time spent in hypoglycemia, and clinically significant hypoglycemia, were summarized with number of participants, mean, SD, median, and interquartile range (IQR). Changes from baseline values were calculated at each time point and summarized descriptively; the exception to this is the time spent with PG within prespecified ranges, which does not have a baseline value. For part 2 efficacy endpoints, continuous and categorical endpoints were presented using summary statistics or frequencies, respectively. No formal testing was performed. For the time to complete weaning off IV glucose, the time to actual hospital discharge, and the time to pancreatectomy, median time including CI and proportion of participants with the event at weeks 1, 2, and 3 were calculated by Kaplan–Meier estimates.

For those undergoing pancreatectomy, outcome assessment after pancreatectomy was not included in the primary analyses. Time to complete weaning off IV glucose and time to hospital discharge were thus censored at the time of pancreatectomy. Safety analyses were based on the safety analysis set, which included all participants who were administered randomized treatment. The FAS was defined as all participants in the safety analysis set who had a valid baseline efficacy assessment. No formal inferential analyses were conducted for safety variables. An independent data monitoring committee was established to conduct regular reviews of the study's safety data.

## Results

Between June 19, 2020, and February 9, 2022, 16 participants were enrolled, of whom 13 were randomized to study treatment. One participant was randomized in error and did not receive the study treatment. The remaining 12 participants received the study treatment in part 1: 7 were assigned to dasiglucagon–placebo and 5 to placebo–dasiglucagon. All participants completed treatment in part 1 and entered part 2. Upon completion of part 2, 11 participants continued into the extension study. One participant discontinued study treatment owing to planned pancreatectomy and entered the follow-up period (Fig. 1B). The study completion date was March 7, 2022.

Baseline demographics and clinical characteristics of the FAS population are shown in Table 1. At study entry, the median (IQR) age was 38.5 (28.0-89.5) days, most participants were male (83.3%), and most had not undergone pancreatectomy (91.7%). The mean (SD) dasiglucagon infusion rate was 57.7 (6.8) μg/hour in part 1 and 44.2 (21.1) μg/hour (week 1), 47.6 (21.4) μg/hour (week 2), and 48.9 (23.0) μg/hour (week 3) in part 2.

**Table 1.** Demographics and baseline characteristics (FAS population)

	Dasiglucagon and placebo (n = 12)
Age, days, median (IQR)	38.5 (28.0-89.5)
Length, cm, median (IQR)	55.9 (54.9-63.0)
Weight, kg, median (IQR)	6.6 (4.6-7.5)
Sex, n (%)	
Male	10 (83.3)
Female	2 (16.7)
Ethnicity, n (%)	
Not Hispanic or Latino	11 (91.7)
Hispanic or Latino	1 (8.3)
Race, n (%)	
White	8 (66.7)
Asian	2 (16.7)
American Indian or Alaska Native	0 (0.0)
Black or African American	0 (0.0)
Native Hawaiian or other Pacific Islander	0 (0.0)
Other	2 (16.7)
Pancreatectomy, n (%)	
None	11 (91.7)
Near-total (>95%)	0 (0.0)
Partial (<95%)	1 (8.3)
Gastrostomy/nasogastric tube, n (%)	
Nasogastric tube	7 (58.3)
None	3 (25.0)
Gastrostomy	2 (16.7)

Abbreviations: FAS, full analysis set; IQR, interquartile range.

**Figure 2.**
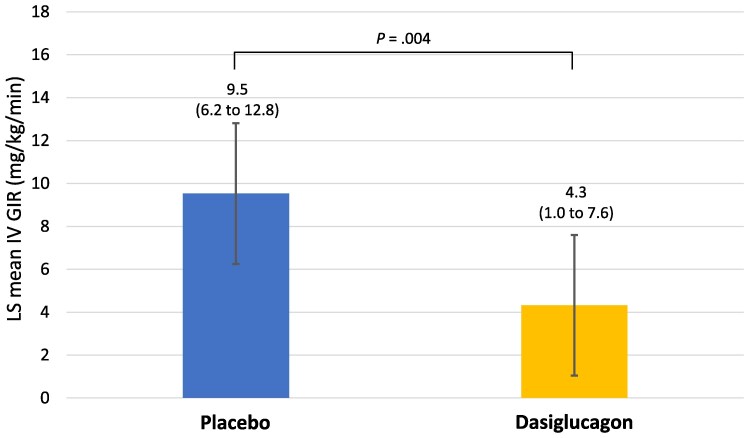
Weighted mean IV GIR over the last 12 hours of the first treatment period during randomized, blinded, crossover study treatment (dasiglucagon or placebo) in part 1 (primary analysis; FAS population). Error bars represent 95% CI.

Abbreviations: CI, confidence interval; FAS, full analysis set; GIR, glucose infusion rate; LS, least-squares.

The primary and key secondary endpoints were met. The least-squares (LS) mean [95% CI] IV GIR was significantly reduced with dasiglucagon (4.3 [1.0 to 7.6] mg/kg/min) compared with placebo (9.5 [6.2 to 12.8] mg/kg/min), with a difference (95% CI) of −5.2 (−8.3 to −2.1) mg/kg/min (*P* = .004) (Fig. 2). During part 1, the total amount of carbohydrates administered, independent of the route, was significantly lower with dasiglucagon than with placebo. The treatment difference between placebo (138.4 g/day [95% CI, 103.4 to 172.9 g/day]) and dasiglucagon (107.4 g/day [95% CI, 72.9 to 141.9 g/day]) was −30.9 g/day (95% CI, −56.8 to −5.1 g/day; *P* = .02).

During open-label treatment (part 2), the median (95% CI) time to complete weaning off IV glucose was 5.8 (1.0 to 7.9) days, with 2 participants’ data being censored from when they underwent pancreatectomy (for focal and diffuse CHI, respectively) at the end of week 3. Seven participants (58.3%) at the end of week 1 and 10 participants (83.3%) at the end of weeks 2 and 3 had weaned off IV glucose (Table 2). Out of the 10 participants, 7 remained weaned off IV glucose at study completion without the need for pancreatectomy.

**Table 2.** Time to complete weaning off IV glucose, time to hospital discharge, and time to pancreatectomy by week in part 2 (FAS population; Kaplan–Meier estimates)

	Dasiglucagon (n = 12)
Time to complete weaning off IV glucose (days), median (95% CI)	5.8 (1.0 to 7.9)
Cumulative number of participants weaned off IV glucose within week, n (%)	
End of week 1	7 (58.3)
End of week 2	10 (83.3)
End of week 3	10 (83.3)
Cumulative number of participants discharged from hospital within week, n (%)	
End of week 1	0 (0.0)
End of week 2	4 (39.4)
End of week 3	4 (39.4)
Cumulative number of participants with pancreatectomy within week, n (%)	
End of week 1	0 (0.0)
End of week 2	1 (8.3)
End of week 3	1 (8.3)

Percentages calculated by Kaplan–Meier estimates.

Abbreviations: CI, confidence interval; FAS, full analysis set.

The median time to hospital discharge and the median time to pancreatectomy could not be calculated (fewer than 50% of participants experienced these events). At the end of part 2, 4 participants (39.4%) had been discharged from the hospital, all after week 2 (Table 2). One participant underwent preplanned near-total pancreatectomy for diffuse disease in week 2. One participant with focal CHI had partial pancreatectomy in week 1 and required a second pancreatectomy in week 3. However, because the pancreatectomy did not reach a 95% cutoff, this surgery was not included in the analysis of time to pancreatectomy (Table 2).

During part 2 of the study, numerical reductions in the median (IQR) percentage of time spent in hypoglycemia were observed, with 7.2% (6.5% to 14.5%) in week 1, 8.8% (1.9% to 13.8%) in week 2, and 5.7% (1.3% to 8.7%) in week 3, as detected by CGM. The median (IQR) percentage of CGM-detected time spent in clinically significant hypoglycemia was 2.1% (1.1% to 4.5%) in week 1, 2.2% (0.1% to 3.1%) in week 2, and 0.9% (0.2% to 2.3%) in week 3.

All participants experienced 1 or more treatment-emergent AEs (TEAEs). The TEAE incidence reported during part 1 was higher with placebo than with dasiglucagon (Table 3); however, owing to the crossover design randomization, 7 participants received dasiglucagon before receiving placebo. During open-label dasiglucagon treatment in part 2, 10 participants (83.3%) had at least 1 TEAE. One participant (8.3%) in each group during part 1 and 5 participants (41.7%) in part 2 experienced a TEAE considered possibly treatment-related. There were no serious TEAEs during part 1; 1 participant (8.3%) had 2 serious TEAEs of acute respiratory failure/respiratory distress during part 2. Neither event was considered to be related to study treatment. No deaths or discontinuations due to TEAEs were reported (Table 3). The most frequently reported TEAEs for both study parts were skin and subcutaneous tissue disorders, gastrointestinal events, and metabolism and nutrition disorders (Table 3).

**Table 3.** AEs reported during the study in part 1 and part 2 (safety population)

	Part 1				Part 2	
	Placebo (n = 12)		Dasiglucagon (n = 12)		Dasiglucagon (n = 12)	
	Participants, n (%)	Events, events/PYE	Participants, n (%)	Events, events/PYE	Participants, n (%)	Events, events/PYE
Safety overview						
TEAEs	7 (58.3)	10/152.2	3 (25.0)	6/91.3	10 (83.3)	60/99.2
Serious TEAEs	0 (0.0)	0/0.0	0 (0.0)	0/0.0	1 (8.3)	2/3.3
TEAEs leading to discontinuation of study treatment	0 (0.0)	0/0.0	0 (0.0)	0/0.0	0 (0.0)	0/0.0
TEAEs possibly related to study treatment*^a^*	1 (8.3)	1/15.2	1 (8.3)	1/15.2	5 (41.7)	9/14.9
AESIs	2 (16.7)	2/30.4	0 (0.0)	0/0.0	3 (25.0)	4/6.6
Most common TEAEs						
AST increased	1 (8.3)	1/15.2	1 (8.3)	1/15.2	2 (16.7)	2/3.3
Vomiting	1 (8.3)	1/15.2	1 (8.3)	1/15.2	2 (16.7)	2/3.3
ALT increased	1 (8.3)	1/15.2	1 (8.3)	1/15.2	1 (8.3)	1/1.7
Anemia	0 (0.0)	0/0.0	0 (0.0)	0/0.0	3 (25.0)	4/6.6
Papular rash	0 (0.0)	0/0.0	0 (0.0)	0/0.0	3 (25.0)	3/5.0
Hypokalemia	1 (8.3)	1/15.2	0 (0.0)	0/0.0	2 (16.7)	3/5.0
Pyrexia	1 (8.3)	1/15.2	0 (0.0)	0/0.0	2 (16.7)	2/3.3
Constipation	0 (0.0)	0/0.0	0 (0.0)	0/0.0	2 (16.7)	2/3.3
Diaper dermatitis	0 (0.0)	0/0.0	0 (0.0)	0/0.0	2 (16.7)	2/3.3
Gastroesophageal reflux disease	0 (0.0)	0/0.0	0 (0.0)	0/0.0	2 (16.7)	2/3.3
Hyponatremia	0 (0.0)	0/0.0	0 (0.0)	0/0.0	2 (16.7)	4/6.6
AESIs						
Suspicion of NME	1 (8.3)	1/15.2	0 (0.0)	0/0.0	3 (25.0)	3/5.0
Rash	1 (8.3)	1/15.2	0 (0.0)	0/0.0	0 (0.0)	0/0.0
Papular rash	0 (0.0)	0/0.0	0 (0.0)	0/0.0	2 (16.7)	2/3.3
Dry skin	0 (0.0)	0/0.0	0 (0.0)	0/0.0	1 (8.3)	1/1.65
Post-dose hemodynamic changes	1 (8.3)	1/15.2	0 (0.0)	0/0.0	1 (8.3)	1/1.7
Tachycardia	1 (8.3)	1/15.2	0 (0.0)	0/0.0	1 (8.3)	1/1.7
Neurological events	0 (0.0)	0/0.0	0 (0.0)	0/0.0	0 (0.0)	0/0.0
Risk of liver injury	0 (0.0)	0/0.0	0 (0.0)	0/0.0	0 (0.0)	0/0.0

Abbreviations: AE, adverse event; AESI, adverse event of special interest; ALT, alanine aminotransferase; AST, aspartate aminotransferase; MedDRA, Medical Dictionary for Regulatory Activities; NME, necrolytic migratory erythema; PYE, patient-years of exposure; TEAE, treatment-emergent adverse event.

^*a*^As assessed by the investigator.

Includes a safety overview, the incidence of TEAEs reported in 2 or more participants in each treatment group by MedDRA preferred term in part 1 and part 2 and the incidence of AESIs by MedDRA AESI type and preferred term.

During part 1, no participant developed AESIs with dasiglucagon but 2 participants (16.7%) developed AESIs with placebo: 1 event of suspicion of NME (rash of mild intensity, not confirmed to be NME after dermatologist evaluation, resolved after 3 weeks) and 1 event of postdose hemodynamic changes (resolving mild tachycardia) (Table 3). During part 2, 3 participants (25.0%) experienced 4 AESIs: 3 events of suspicion of NME, all of which were not confirmed to be NME after dermatologist evaluation (1 event of dry skin of mild intensity, which resolved at trial completion; 2 separate events of resolving mild papular rash, with 1 event assessed as possibly treatment-related) and 1 event of postdose hemodynamic changes (self-resolving tachycardia with coexisting anemia and fever) (Table 3). No participant developed AESIs of risk of liver injury, loss of consciousness, or partial and generalized seizures (Table 3). No remarkable abnormalities were found in the clinical and laboratory assessments. Among the participants tested (n = 10, 2 participants did not have samples collected), none had ADA-positive results. Overall, 13 technical complaints were reported in 7 participants, including for the study devices.

## Discussion

In this double-blind, placebo-controlled, randomized crossover study in newborns and infants with recently diagnosed CHI and dependent on IV glucose, dasiglucagon treatment resulted in a statistically significant reduction of IV glucose and total carbohydrate requirement. The decrease in mean IV GIR with dasiglucagon represents a 55% reduction compared with placebo. Open-label dasiglucagon allowed most participants to be weaned off IV glucose with clinically acceptable CGM glucose profiles. In a patient cohort often requiring pancreatectomy, only 2 out of 12 participants had pancreatectomy during the study. Of the remaining 10 participants, 7 remained completely weaned off IV glucose without the need for pancreatectomy, and 4 participants were discharged home from the hospital by the end of a 25-day observation period.

Overall, dasiglucagon was well-tolerated and no unusual safety concerns were identified. The most frequently reported TEAEs were known class effects (15) and consistent with a previous study (13). Skin AEs were reported more frequently with dasiglucagon than with placebo, with 3 events of suspicion of NME without confirmation. These AEs are consistent with known class effects (16) and constitute the most relevant side effects of dasiglucagon treatment. No serious TEAEs were reported during part 1; 1 participant experienced 1 serious TEAE during part 2, unrelated to dasiglucagon. All but 1 participant continued dasiglucagon in the safety extension study.

Dasiglucagon treatment led to numerical reductions in the proportion of time spent in hypoglycemia in infants with severe CHI at high risk of neurodevelopmental sequelae and often requiring pancreatectomy. Importantly, during open-label treatment, dasiglucagon-mediated glycemic stability may have facilitated early discharge from the hospital and contributed to the avoidance of pancreatectomy, making dasiglucagon a promising medical treatment option for children at the most severe end of the CHI spectrum.

Dasiglucagon is stable in liquid formulation and suitable for long-term home treatment by SC infusion; therefore, dasiglucagon could be used as an addition or alternative to standard-of-care therapy, without recourse to hospital admissions or constraining continuous nutritional support and associated complications such as oral aversion, while being aware of potential pump-related technical and operational errors.

Potential limitations of this study are the short duration of the placebo-controlled treatment period (part 1) and the limited number of participants. However, considering that CHI carries a high risk of neurodevelopmental sequelae if treatment is delayed, it was considered unethical for participants at risk of neuroglycopenia to receive the placebo for longer than necessary. Furthermore, a power calculation was undertaken to determine the minimally required number of participants in order to meet the primary endpoint, while at the same time minimizing the number of children who received the placebo. As the primary endpoint was met, the limited number of participants can thus not be considered a weakness for the conclusions on the primary endpoint. Another limitation is the lack of a comparator in part 2. However, a single-arm approach is justified to provide clinical benefit to all participants in a rare disease trial (17). This study did not include children with CHI older than 1 year with the intention to determine the safety and efficacy of dasiglucagon in the most vulnerable patient population, complementing its sister study in children aged 3 months to 12 years (13).

In conclusion, dasiglucagon significantly reduced the IV glucose and carbohydrate requirement in children with severe CHI compared with placebo, reducing the need for pancreatectomy. The study data indicate that dasiglucagon is an efficacious and well-tolerated treatment for young children with CHI. Following completion of the clinical development program, regulatory approval of dasiglucagon in CHI would provide a new therapy option for a rare disease with a high unmet need.

## Data Availability

Restrictions apply to the availability of some or all data generated or analyzed during this study to preserve patient confidentiality or because they were used under license. The corresponding author will on request detail the restrictions and any conditions under which access to some data may be provided.

## Clinical Trial Information

EU Clinical Trials Register: 2017-004545-24; ClinicalTrials.gov: NCT04172441

## Abbreviations

ADA: antidrug antibody
AE: adverse event
AESI: adverse event of special interest
CGM: continuous glucose monitoring
CHI: congenital hyperinsulinism
CI: confidence interval
FAS: full analysis set
GIR: glucose infusion rate
IQR: interquartile range
LS: least-squares
NME: necrolytic migratory erythema
PG: plasma glucose
SC: subcutaneous
TEAE: treatment-emergent adverse event
